# Modeling Shear-Thinning Flow in Twin-Screw Extrusion Processes

**DOI:** 10.3390/pharmaceutics17030353

**Published:** 2025-03-09

**Authors:** Vincent Kimmel, Lorena Gräfe, Luca Grieser, Alexey Lips, Robert Hennig, Judith Winck, Markus Thommes

**Affiliations:** 1 Laboratory of Solids Process Engineering, Department of Biochemical and Chemical Engineering, Technical University Dortmund, Emil-Figge-Str. 68, 44227 Dortmund, Germany; vincent.kimmel@tu-dortmund.de (V.K.); lorena.graefe@tu-dortmund.de (L.G.); luca.grieser@tu-dortmund.de (L.G.); alexey.lips@tu-dortmund.de (A.L.); judith.winck@tu-dortmund.de (J.W.); 2 Drug Delivery Innovation Center, INVITE GmbH, Chempark Building W32, Otto-Bayer-Str. 32, 51061 Cologne, Germany; 3 Global Drug Product Development, Global CMC Development, Merck Healthcare KGaA, Frankfurter Str. 250, 64293 Darmstadt, Germany; robert.hennig@merckgroup.com

**Keywords:** twin-screw extruder, screw characteristics, one-dimensional modeling, A and B parameters, mechanistic modeling, shear thinning

## Abstract

**Background/Objective:** Hot-melt extrusion has been established as a formulation strategy for various pharmaceutical applications. However, tailoring the screw configuration is a major challenge where 1D modeling is utilized. This usually requires specific screw parameters, which are rarely noted in the literature, especially when dealing with shear-thinning formulations. **Methods:** Therefore, a custom-made test rig was used to assess the behavior of various conveying and kneading elements using Newtonian silicon oil and shear-thinning silicon rubber. The pressure and the power were measured as a function of volume flow. A model was proposed characterizing the screw element behavior by six individual parameters $\left(A_{1},\;A_{2},\;A_{3},\;B_{1},\;B_{2},\;B_{3}\right)$. **Results:** The experimental results regarding the behavior with respect to Newtonian fluids were in good agreement with the literature and were modeled in accordance with the Pawlowski approach. In terms of shear-thinning fluids, the influence of screw speed on pressure and power was quantified. An evaluation framework was proposed to assess this effect using two additional parameters. Based on a high number of repetitive measurements, a confidence interval for the individual screw parameters was determined that is suitable to highlight the differences between element types. **Conclusions:** Finally, geometrical screw parameters for Newtonian and shear-thinning flow were assessed and modeled, with three conveying and three kneading elements characterized.

## 1. Introduction

Extrusion processes are established in the plastic and food industries for processing highly viscous materials. In recent years, the extrusion process has also been increasingly utilized in pharmaceutical applications [1,2,3]. Normally, intermeshing co-rotating twin-screw extruders are used due to their high mixing performance, self-cleaning properties, continuous processing, and modular equipment design [4,5]. The latter is achieved by using different conveying or kneading screw elements, which are assembled in specific sequences to account for the formulation and process needs [6].

**Figure 1:**
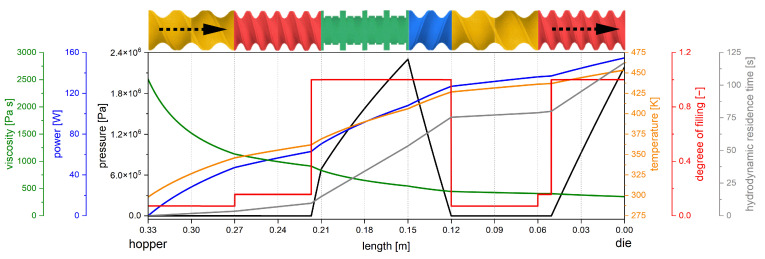
Schematic of a 1D model for an entire extrusion process. A generic screw configuration with various elements is given at the top and the graphical representation of pressure, power, viscosity, temperature, degree of filling, and residence time is shown below [20].

A tailored screw design for a specific drug product cannot normally be assessed iteratively using experiments. The high number of screw elements in a screw, as well as the various screw types, result in an incredibly high number of potential combinations. Therefore, modeling approaches are required to enable in silico screening to reduce development time and shorten the time to market. To achieve this, 3D simulations are often proposed using substance and equipment data. These offer high spatial resolution, where different concepts such as smoothed particle hydrodynamics [7,8,9], the finite element method [10,11,12] and the finite volume method [13] are used. The disadvantage of these methods is the high computational effort, which makes the screw sequence screening quite slow. In 1D modeling, the extruder is modeled in the axial direction, typically ignoring radial effects. This approach is not as universal as 3D simulations since some processes in the screw are not captured adequately [14,15]. However, it is fast and able to predict several process conditions and material attributes axially from the hopper to the die (Figure 1) within a few seconds [16,17,18,19].

There are several 1D software packages for modeling twin-screw extrusion, including Akro-Co Twin-Screw [21,22], Sigma [23,24], and Ludovic [17,25]. However, the modeling approach used is sometimes not clear, and the algorithms are usually not disclosed. This reduces scientific value and avoids any further development of these software applications by the scientific community. Therefore, a mechanistic approach of Pawlowski [26] was considered in this study which is well described in the literature [6,18] but relies on specific screw parameters based on dimensionless numbers that are not in the public domain.

The aim of this study is to establish an experimental and modeling framework to determine screw parameters using Newtonian and shear-thinning materials. Therefore, common conveying and kneading elements were characterized in a test rig and dimensionless parameters were utilized to describe the individual performance. These will serve as the origin of 1D modeling capable of performing in silico screw optimization.

## 2. Theoretical Framework

The individual screw parameters used in this study were determined in accordance with the approach from Pawlowski [26], which was developed initially for single-screw extruders but later extended to twin-screw extruders [6]. It is based on three dimensionless quantities. The dimensionless volume flow $\left({\dot{V}}^{*}\right)$ is defined as the volumetric throughput $\left(\dot{V}\right)$ over the screw speed $\left(n\right)$ and the cube of the barrel diameter $\left(d\right)$ in Equation (1) [27].

(1) $${\dot{V}}^{*}=\frac{\dot{V}}{n\cdot d^{3}}$$

Dimensionless pressure $\left({\Delta p}^{*}\right)$ is obtained by the pressure gradient $\left(∆p\right)$ along the axial length $\left(l\right)$ and depends on the barrel diameter $\left(d\right)$, the screw speed $\left(n\right)$, and the viscosity $\left(\eta \right)$ in Equation (2) (left) [27].

(2) $${\Delta p}^{*}=\frac{∆p\cdot d}{l\cdot n\cdot \eta }=-\frac{A_{2}}{A_{1}}\cdot {\dot{V}}^{*}+A_{2}$$

**Figure 2:**
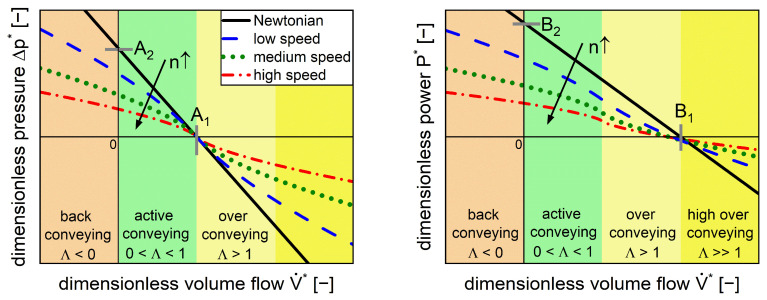
Schematic of pressure characteristic (**left**) and power characteristic (**right**) for Newtonian flow behavior (black lines) and shear-thinning flow (colored lines). Determination of the dimensionless parameters $\left(A_{1},A_{2},B_{1},B_{2}\right)$ is based on the axis intercepts. The process regimes are classified as back conveying (orange), active conveying (green), over conveying (light yellow), and high over conveying (dark yellow). The arrows represent an increase in screw speed $\left(n\right)$ [6].

A linear correlation between dimensionless pressure and dimensionless volume flow for Newtonian, isothermal, laminar, single-phase, and fully filled extrusion screw conditions (Figure 2, left, black line) can be described with the dimensionless pressure parameters $\left(A_{1},A_{2}\right)$ (Equation (2), right) [26]. These parameters depend solely on the screw geometry (diameters, pitch) and correspond to the intercepts with the axes. Specifically, $A_{1}$ is defined as the dimensionless inherent throughput $\left({\Delta p}^{*}=0\right)$ and $A_{2}$ is defined as the maximal dimensionless pressure build-up $\left({\dot{V}}^{*}=0\right)$ [6].

The conveying parameter $\left(\Lambda \right)$ in Equation (3) is used to classify the process regimes into the back pumping, overrun, and active conveying sections (Figure 2).

(3) $$\Lambda =\frac{{\dot{V}}^{*}}{A_{1}}$$

The dimensionless power $\left(P^{*}\right)$ in Equation (4) (left) is defined as the ratio of the power, which is described through the torque $\left(M\right)$ multiplied by the peripheral speed $\left(2\cdot \pi \cdot n\right)$ of the screws, to the product of the axial length $\left(l\right)$, the screw speed $\left(n\right)$ squared, the barrel diameter $\left(d\right)$ squared, and the viscosity $\left(\eta \right)$ [27].

(4) $$P^{*}=\frac{M\cdot 2\cdot \pi \cdot n}{l\cdot n^{2}\cdot d^{2}\cdot \eta }=-\frac{B_{2}}{B_{1}}\cdot {\dot{V}}^{*}+B_{2}$$

A linear relation (Figure 2, right, black line) under the abovementioned boundary conditions for the power characteristics is described by the dimensionless power parameters $\left(B_{1},\;B_{2}\right)$ (Equation (4), right). Here, the parameter $B_{1}$ is defined as the turbine point, where the energy transfer changes from the fluid to the screw and $B_{2}$ is the dimensionless power input for a closed extrusion die $\left({\dot{V}}^{*}=0\right)$ [6]. These parameters for the pressure characteristics $\left(A_{1},\;A_{2}\right)$ and the power characteristics $\left(B_{1},\;B_{2}\right)$ can either be generated by experiments or 3D simulations. The validity of this model for Newtonian fluids has already been demonstrated in simulations and in experiments [10,28,29].

For the shear-thinning behavior, the Carreau–Arrhenius [30] viscosity model was utilized. There, the temperature influence on viscosity is captured by a shift factor $\left(a_{T}\right)$ (Equation (5)) [31].

(5) $$a_{T}\left(T,T_{ref}\right)=exp\left(\frac{E_{A}}{R}\cdot \left(\frac{1}{T}-\frac{1}{T_{ref}}\right)\right)$$

The influence of shear rate $\left(\dot{\gamma }\right)$ on dynamic viscosity $\left(\eta \right)$ depends on three parameters, the zero shear rate viscosity $\left(\eta_{0}\right)$, the critical shear rate $\left({\dot{\gamma }}_{c}\right)$, and the flow index $\left(c\right)$, as well as the temperature shift factor $\left(a_{T}\right)$ (Equation (6)) [30].

(6) $$\eta \left(\dot{\gamma },T\right)=\frac{\eta_{0}\cdot a_{T}}{{\left(1+a_{T}\cdot \frac{\dot{\gamma }}{{\dot{\gamma }}_{c}}\right)}^{c}}$$

The shear-thinning viscosity model (Equation (6)) can be combined with the pressure model (Equation (2)) to describe the pressure characteristic of a shear-thinning material (Equation (7)) [6].

(7) $${\Delta p}^{*}=\frac{∆p\cdot d}{l\cdot n\cdot \eta_{0}\cdot a_{T}}=\frac{A_{2}\cdot \left(1-\frac{{\dot{V}}^{*}}{A_{1}}\right)}{{\left(1+a_{T}\cdot \frac{\dot{\gamma }}{{\dot{\gamma }}_{c}}\right)}^{c}}$$

However, the specific shear rate $\left(\dot{\gamma }\right)$ in the screw element is unknown but is related to the screw speed (n). Therefore, a representative shear rate $\left({\dot{\gamma }}_{r}\right)$ was introduced using throttling $\left(1-\Lambda \right)$ as well as a screw-specific correlation factor $\left(A_{3}\right)$ in Equation (8) [32].

(8) $${\dot{\gamma }}_{r}=A_{3}\cdot n\cdot \left(1-\Lambda \right)$$

The dimensionless shear parameter, $A_{3}$, is only dependent on the screw geometry, similar to $A_{1}$ and $A_{2}$. It describes how the screw geometry influences the shear rate for the pressure characteristics using a shear-thinning fluid (Equation (9)).

(9) $${\Delta p}^{*}=\frac{∆p\cdot d}{l\cdot n\cdot \eta_{0}\cdot a_{T}}=\frac{A_{2}\cdot \left(1-\frac{{\dot{V}}^{*}}{A_{1}}\right)}{{\left(1+a_{T}\cdot A_{3}\cdot n\cdot \left(1-\frac{{\dot{V}}^{*}}{A_{1}}\right)\cdot \frac{1}{{\dot{\gamma }}_{c}}\right)}^{c}}$$

While the dimensionless pressure correlates linearly with the dimensionless volume flow for a Newtonian fluid (Figure 2, left, black line), a nonlinear behavior was observed for the shear-thinning fluid. Moreover, higher screw speeds led to lower dimensionless pressure (Figure 2, left, colored dashed lines). At dimensionless inherent throughput $\left({\dot{V}}^{*}=A_{1},\;{∆p}^{*}=0\right)$, all functions merge since the representative shear rate converges to zero, meaning that the zero viscosity applies at all screw speeds. In addition, the Newtonian fluid touches this point since the viscosity is included in the dimensionless pressure.

The dimensionless power (Figure 2, right) includes both the power associated with pressure build-up, which is linked to throughput (pressure-flow power), and the power associated with agitating the material in the extruder, which is related to screw speed (drag-flow power). Both terms also account for the power required to process the material; for example, the power required for heating or mixing. At dimensionless inherent throughput (${\dot{V}}^{*}=A_{1}$, intersection yellow and green shaded areas) the dimensionless pressure is zero and the power is solely related to drag flow. The shear-thinning behavior of the material leads to lower dimensionless power with an increase in screw speed. The functions of the power characteristic do not necessarily merge in one point.

In order to describe the dimensionless power characteristics for shear-thinning fluids, an additional parameter, $B_{3}$, is required, which captures the shear-thinning effects independent of the throughput. This correlates a second representative shear rate to screw speed and is independent from throttling. Therefore, the product of $B_{3}$ and n is inserted into the Carreau–Arrhenius model (Equation (6)), implementing a second representative shear rate.

(10) $$P^{*}=\frac{P}{l\cdot n^{2}\cdot d^{2}\cdot \eta_{0}\cdot a_{T}}=\frac{B_{2}\cdot \frac{A_{1}}{B_{1}}\cdot \left(1-\frac{{\dot{V}}^{*}}{A_{1}}\right)}{{\left(1+a_{T}\cdot A_{3}\cdot n\cdot \left(1-\frac{{\dot{V}}^{*}}{A_{1}}\right)\cdot \frac{1}{{\dot{\gamma }}_{c}}\right)}^{c}}+\frac{B_{2}\cdot \left(1-\frac{A_{1}}{B_{1}}\right)}{{\left(1+a_{T}\cdot B_{3}\cdot n\cdot \frac{1}{{\dot{\gamma }}_{c}}\right)}^{c}}$$

Equations (2), (6) and (9) are combined in Equation (10) to give the dimensionless power for a shear-thinning fluid. Thereby, the term to the left of the addition operator is related to pressure flow while the term on the right is the power shear flow. Finally, the dimensionless shear parameters $\left(A_{3},B_{3}\right)$ are mandatory to describe shear-thinning behavior in twin-screw extrusion processes.

## 3. Materials and Methods

### 3.1. Materials

Silicone oil (Newtonian fluid, Bluesil FLD 47V100000, Elkem Silicones, Lübeck, Germany), liquid silicone rubber (shear-thinning fluid, Elastosil LR 3003/10 B, Wacker AG, Stuttgart, Germany), and a solution of water and 3 wt% hydroxyethylcellulose (HEC) (shear-thinning fluid, Natrosol 250 M Pharm, Ashland Industries Europe, Schaffhausen, Switzerland) were utilized.

### 3.2. Rheology Measurement Method

The rheological measurements were performed in a rotational rheometer (Haake Mars 60, Thermo Fischer Scientific, Karlsruhe, Germany) equipped with a cup-rotor geometry (CCB25, Thermo Fischer Scientific, Karlsruhe, Germany) with 17 mL sample volume and a gap of 5.3 mm. The triplicate measurements were performed in controlled shear rate mode in a range from 0.01 to 100 $s^{-1}$ at temperatures of 20, 25, and 30 °C. The data were evaluated according to the Carreau–Arrhenius approach [33].

### 3.3. Extrusion Screw Test Rig Method

**Figure 3:**
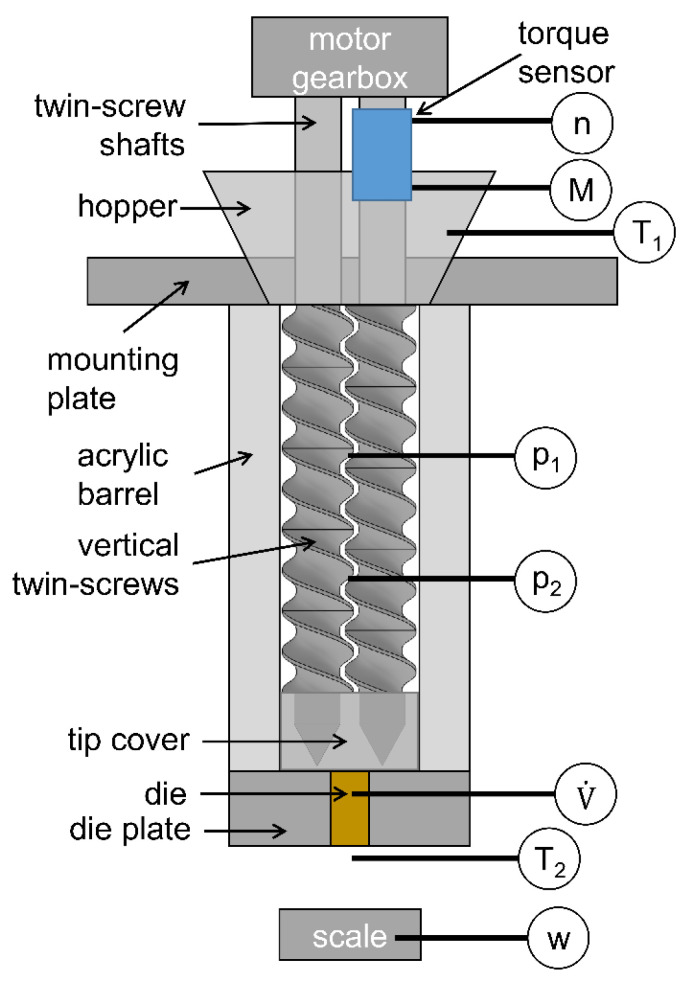
Schematic of the extrusion screw test rig for the characterization of pressure and power. Vertically arranged twin screws with probes for speed, torque, temperature, pressure, and mass flow. The volume flow is adjusted by the die diameter.

The individual extrusion screw elements were characterized in a specially designed test rig. This was geometrically similar to the Leistritz ZSE 27 MAXX extruder (Leistritz, Nürnberg, Germany). The twin screws were vertically arranged to guarantee complete filling of the barrel, which was required to apply the models. The vertical orientation enabled air bubbles to rise to the top, resulting in a homogeneous, single-phase fluid (Figure 3).

A torque sensor was placed directly on one screw shaft to accurately measure the screw speed $\left(n\right)$ and the torque $\left(M\right)$. Afterwards, a hopper was placed at the top where the fluid was filled, a constant fill level was maintained, and the temperature $\left(T_{1}\right)$ was measured to account for viscosity changes. The transparent acrylic barrels offer multiple ports at different axial positions for the pressure probes $\left(p_{1}\right)$ and $\left(p_{2}\right)$. Dies with different diameters (1, 2, 3, 4, 5, 6, and 12.5 mm) were mounted in the die plate to adjust the volume flow $\left(\dot{V}\right)$. When the fluid left the extruder, a second temperature probe, $T_{2}$, was used to check on deviations from the isothermal conditions. In all measurements, the extruder test rig was equipped with one type of screw element. The process data of speed, torque, temperature, pressure, and mass flow were measured over time in a steady state.

## 4. Results and Discussion

### 4.1. Rheology of Model Fluids

**Figure A1:**
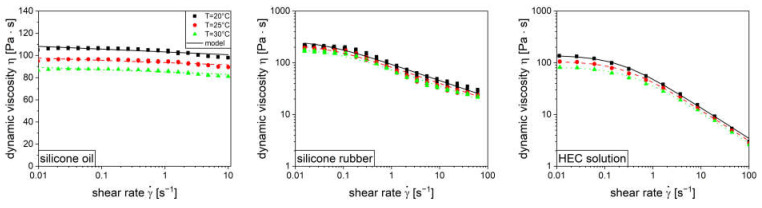
Flow curves for the rheological characterization by dynamic viscosity over shear rate for silicone oil (**left**, linear *y*-axis), silicone rubber (**center**, logarithmic *y*-axis), and HEC solution (**right**, logarithmic *y*-axis).

Three model fluids were chosen for this study in order to characterize screw elements with respect to Newtonian and shear-thinning material behavior. The origin of the material selection was a previous study [33] where common polymers used in hot-melt extrusion were characterized. The utilized model fluids aimed to exhibit similar rheological behavior but at room temperature. Furthermore, the operating conditions of the test rig were considered as well, allowing a wide range of process conditions. First investigations dealt with the rheological characterization of these materials, considering different shear rates as well as temperatures. The flow functions are given in the Appendix A (Figure A1) and the parameters of the Carreau–Arrhenius model (Equations (5) and (6)) are shown in Table 1.

The silicone oil exhibited Newtonian behavior while the silicone rubber and the aqueous hydroxyethylcellulose solution (HEC solution) exhibited shear-thinning behavior. Overall, the rheological behavior of these materials at room temperature was considered to be comparable to polymer melts at elevated temperatures in hot-melt extrusion [5,37].

### 4.2. Selected Screw Elements

**Figure 4:**
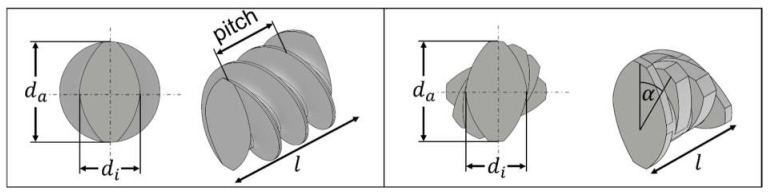
Common types of screw elements of a twin-screw extruder: conveying elements with a pitch of 20 mm (**left**) and a kneading element with a staggering angle of 30° (**right**), including relevant geometric parameters like the outer diameters $\left(d_{a}\right)$, inner diameter $\left(d_{i}\right)$, length $\left(l\right)$, pitch, and staggering angle $\left(\alpha \right)$.

In pharmaceutical extrusion processes, conveying (Figure 4, left) as well as kneading elements (Figure 4, right) are frequently used and exhibit different transport and mixing capacities [38]. Moreover, three different conveying (pitch: 20, 30, 40 mm) and three kneading elements (staggering angle: 30, 60, 90°) were used, reflecting a wide range of configurations. Conveying elements were selected with consideration of the outer diameter $\left(d_{a}\right)$, the ratio to the inner diameter $\left(d_{i}\right)$, the element length $\left(l\right)$, and the pitch (Figure 4, left). Additionally, the kneading elements were selected according to the staggering angle $\left(\alpha \right)$ rather than by using the pitch (Figure 4, right).

Throughout this manuscript, the conveying elements are named GFA followed by the pitch in mm (e.g., GFA-20), while the kneading elements are abbreviated as KB followed by the staggering angle (e.g., KB-30). The exact dimensions of the extruder barrel and the screw elements are given in Table A1.

### 4.3. Optimization of Extrusion Test Rig

The extruder test rig that was used was developed in a previous study [28]. However, some modifications were made. Specifically, the mechanical power of the electric motor was increased in order to extend the operating range, the alignment of the screws to the extrusion barrel was optimized to minimize its contact and improve torque readings, and the temperature sensors at the inlet and outlet were used to correct for viscosity changes based on temperature.

**Figure 5:**
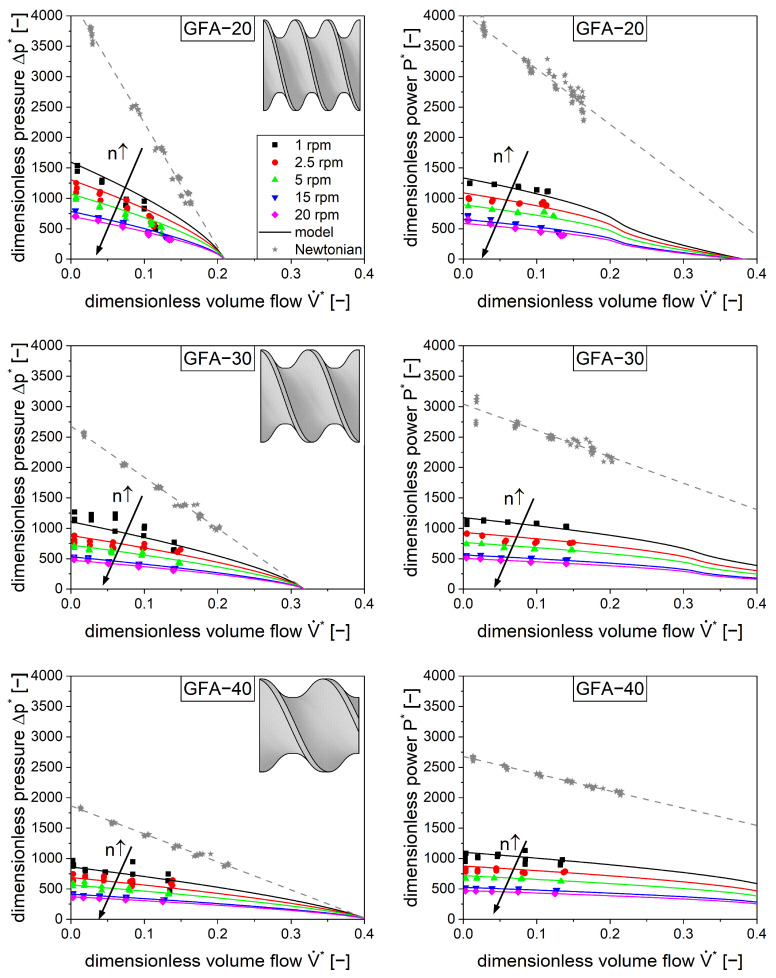
Measured dimensionless pressure (**left**) and power (**right**) characteristics for conveying elements with a pitch of 20 mm (**top**), 30 mm (**center**), and 40 mm (**bottom**) for shear-thinning flow (colored symbols) with silicone rubber and Newtonian flow (gray stars) with silicone oil. The lines represent the model (Equations (9) and (10)). The arrows represent an increase in screw speed $\left(n\right)$.

Based on these modifications, the pressure and power characteristics were measured using the Newtonian fluid (silicone oil). The screw speed and die diameter were altered systematically and the volume flow, pressure difference, and power were measured. The data were evaluated by linear regression (Figure 5, gray symbols and line). Overall, the pressure characteristics are in good agreement with the data from a previous work [28]. However, the power consumption is significantly lower than in previous experiments. This was attributed to less friction between the screw elements and the barrel due to the better alignment of these components. The current power data were in much better agreement with the simulation data of König [6].

### 4.4. Characterization of Conveying Elements

The pressure and power characteristics of different conveying elements for shear-thinning fluids were measured using silicone rubber as the model compound. As with the Newtonian fluid, the die diameter and the screw speed were altered systematically, and the volume flow, pressure difference, and power were measured. Generally, the shear-thinning material (Figure 5, colored symbols) showed much lower pressure and power values compared to the Newtonian material (Figure 5, gray stars), which is attributed to the decrease in viscosity with an increase in shear rate. This phenomenon is also responsible for the further decrease in pressure and power with an increase in screw speed $\left(n↑\right)$. It is also worth noting the nonlinear influence of the screw speed on the pressure difference and power, which is attributed to the complex rheological behavior of the material.

In order to model the pressure and the power for shear-thinning fluids, two parameters, $A_{3}$ and $B_{3}$, are required, as previously derived. To assess these values based on the experimental data, Equations (9) and (10) were linearized, resulting in Equations (11) and (12), and a linear regression was applied.

(11) $${\left(\frac{{∆p}^{*}}{A_{2}\cdot \left(1-\frac{{\dot{V}}^{*}}{A_{1}}\right)}\right)}^{-\left(\frac{1}{c}\right)}-1=A_{3}\cdot a_{T}\cdot n\cdot \left(1-\frac{{\dot{V}}^{*}}{A_{1}}\right)\cdot \frac{1}{{\dot{\gamma }}_{c}}$$

(12) $${\left(\begin{array}{c} \;\\ \frac{P^{*}-\frac{B_{2}\cdot \frac{A_{1}}{B_{1}}\cdot \left(1-\frac{{\dot{V}}^{*}}{A_{1}}\right)}{{\left(1+{a_{T}\cdot A}_{3}\cdot n\cdot \left(1-\frac{{\dot{V}}^{*}}{A_{1}}\right)\cdot \frac{1}{{\dot{\gamma }}_{c}}\right)}^{c}}}{B_{2}\cdot \left(1-\frac{A_{1}}{B_{1}}\right)} \end{array}\right)}^{-\left(\frac{1}{c}\right)}-1=B_{3}\cdot a_{T}\cdot n\cdot \frac{1}{{\dot{\gamma }}_{c}}$$

**Figure 6:**
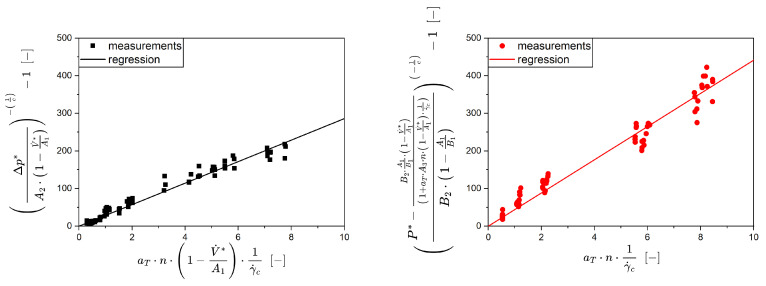
Linearized Equation (11) for pressure (**left**) and linearized Equation (12) for power (**right**) characteristics for the determination of the characteristic shear parameters as the slope of the origin lines. Exemplary for the conveying element GFA-30.

The slope of both functions includes the model parameters $\left(A_{3},B_{3}\right)$ along with several constants and known parameters $\left({\dot{V}}^{*},{∆p}^{*},P^{*},n\right)$. For this kind of data evaluation, the parameters for the Newtonian behavior ($A_{1},A_{2}$ and $B_{1},B_{2}$) as well as the rheological behavior are required as input variables. Figure 6 shows a clear example of a linear regression for the GFA-30 element.

Equations (11) and (12) are able to describe the experimental data. A linear correlation can be found for the pressure difference as well as the power, having coefficients of determination of 0.993 and 0.992, respectively. There is a slightly higher variability in the power data which is presumably related to the extremely low torque (0.07 to 0.6 Nm).

The pressure and power characteristics were modeled (Figure 5, colored lines) based on these data. These calculations included the model parameters of the Newtonian measurements ($A_{1},\;A_{2}$ and $B_{1},\;B_{2}$) as well as the parameters for the shear-thinning behavior $\left(A_{3},\;B_{3}\right)$. The model was capable of describing the experimental data (Figure 5, colored symbols) for all three conveying elements.

When comparing the screw parameter to the literature (Table 2), it is worth noting that the data set is extremely small. Two other available studies were considered: König [6], which uses simulations, and Eitzlmayr [29], which uses experiments. Even here, a direct comparison is not possible since clearances between the extruder barrel and screw were not given, which has an influence on the results. Given this, the data from this study are in line with the literature—the magnitude of the parameters is the same and the trends between the screw elements are comparable.

Finally, the individual screw parameters were suitable for quantifying the performance of the conveying elements with respect to transport, pressure build-up, and power consumption. An increase in pitch increases the transport capacity $\left(A_{1}\right)$ but lowers the pressure build-up $\left(A_{2}\right)$. Lower pitches usually lead to more power consumption based on higher pressures, but also due to higher energy dissipation. Elements with a lower pitch are more affected by shear-thinning in terms of pressure flow $\left(A_{3}\right)$ as well as drag flow $\left(B_{3}\right)$.

Generally, the specific screw parameters such as $A_{3}$ and $B_{3}$ should be independent of the material used. Therefore, additional investigations were performed for verification using an aqueous solution of HEC. Comparable screw parameters were found for the three conveying elements, GFA-20, GFA-30, and GFA-40, with $A_{3}$ values of 39.56 ± 2.03, 27.33 ± 1.25, and 21.15 ± 1.17, respectively, and $B_{3}$ values of 79.41 ± 5.15, 43.69 ± 2.66, and 38.53 ± 1.84, respectively. It is worth noting that the HEC solution led to handling difficulties due to the evaporation of water, which caused viscosity changes that resulted in higher variability during the measurements.

### 4.5. Kneading Elements

**Figure 7:**
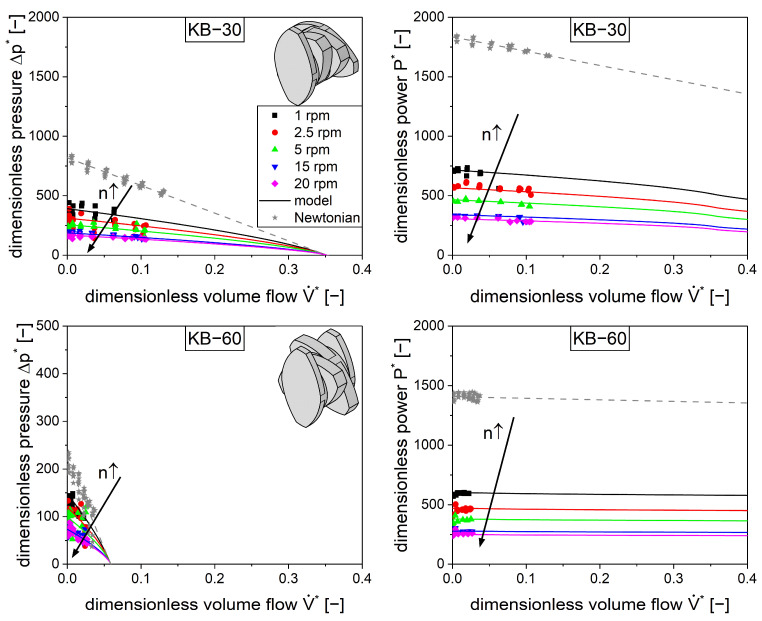
Measured dimensionless pressure (**left**) and power (right) characteristics for kneading elements with a staggering angle of 30° (**top**) or 60° (**bottom**) for shear-thinning flow (colored symbols) with silicone rubber and Newtonian flow (gray stars) with silicone oil. The lines represent the models (Equations (9) and (10)). The arrows represent an increase in screw speed $\left(n\right)$.

In addition to the conveying elements, kneading elements are also commonly used in extrusion processes. Therefore, three elements were chosen with different staggering angles of 30, 60, and 90°. The pressure and power characteristics were determined similarly to the conveying elements using a Newtonian fluid (silicone oil) and a shear-thinning material (silicone rubber). During the experiment, the kneading block with a 90° staggering angle (KB-90) did not show any transport capacity, which is consistent with the literature [39]. Because it was impossible to evaluate the pressure and power characteristics due to no dimensionless volume flow, this element type is not depicted in Figure 7.

Generally, the kneading elements exhibit lower pressure and power values compared to the conveying elements [40,41]. The transport capacity is much lower as can be seen with respect to the dimensionless volume flow due to the staggering of the kneading discs, so the material is less forced in an axial direction. This leads to less power consumed by pressure flow, but the power consumption of the shear flow remains comparatively high. Technically, the kneading block with a staggering angle of 30° is closest to the conveying elements with respect to its geometry. It can be considered a conveying element with a stepwise change in pitch. Therefore, this element is closest to the conveying elements with respect to its performance.

The measurements were carried out using a Newtonian and a shear-thinning fluid where the aforementioned parameters $A_{1},\;A_{2},\;A_{3}$ and $B_{1},\;B_{2},\;B_{3}$ were derived. The model (Figure 7, lines) introduced for the conveying elements is capable of describing the experimental data (Figure 7, symbols) for the kneading elements with staggering angles of 30° and 60° as well.

The comparison of the screw parameters for the individual kneading blocks to the literature is even more challenging than for the conveying elements, because the data set is extremely small (Table 3). There, Stritzinger [10] conducted a simulation while Eitzlmayr [29] performed an experimental investigation. Considering the low quantity of data available, the presented measurements are consistent with the literature.

The kneading elements using a staggering angle of 90° did not have any dimensionless inherent throughput nor pressure build-up. Therefore, the pressure parameters ($A_{1},\;A_{2},\;A_{3})$ were set to zero based on theoretical considerations. Similarly, the turbine point $\left(B_{1}\right)$ was assumed to converge to infinity because high volume flows will not transfer any momentum to the screw. Physically, the 1D model (Equations (9) and (10)) does not fail to describe the kneading block with a 90° staggering angle; it is a special case where the experimental determination of the parameters failed. In terms of the 90° staggering angle, the dimensionless power is independent from the dimensionless volume flow. However, since there is no pressure build-up, the energy is dissipated by drag flow only. Because of the entirely filled barrel, the same quantity of material is stirred independent of the volume flow.

### 4.6. Utilization of the Screw Parameters

The screw parameters ($A_{1},\;A_{2},\;A_{3})$ and ($B_{1},\;B_{2},\;B_{3})$ are meant to be used in 1D modeling in order to identify promising screw configurations for a specific application. A 1D screw sequence screening is beyond the scope of this study, but some general calculations are provided to highlight the value of these investigations. In pharmaceutical applications, hot-melt extrusion is mainly used as a continuous mixing process to disperse or to dissolve a drug substance in a highly viscous polymer melt. In these cases, the throughput given as mass flow rate and the mechanical power are the main relevant variables. The mechanical power is used to force the material through a die but is also dissipated in the viscous melt. The latter is utilized for dispersing and dissolving processes. In this respect, higher power dissipation is often related to higher processability, such as mixing capacity, and is usually desired.

**Figure 8:**
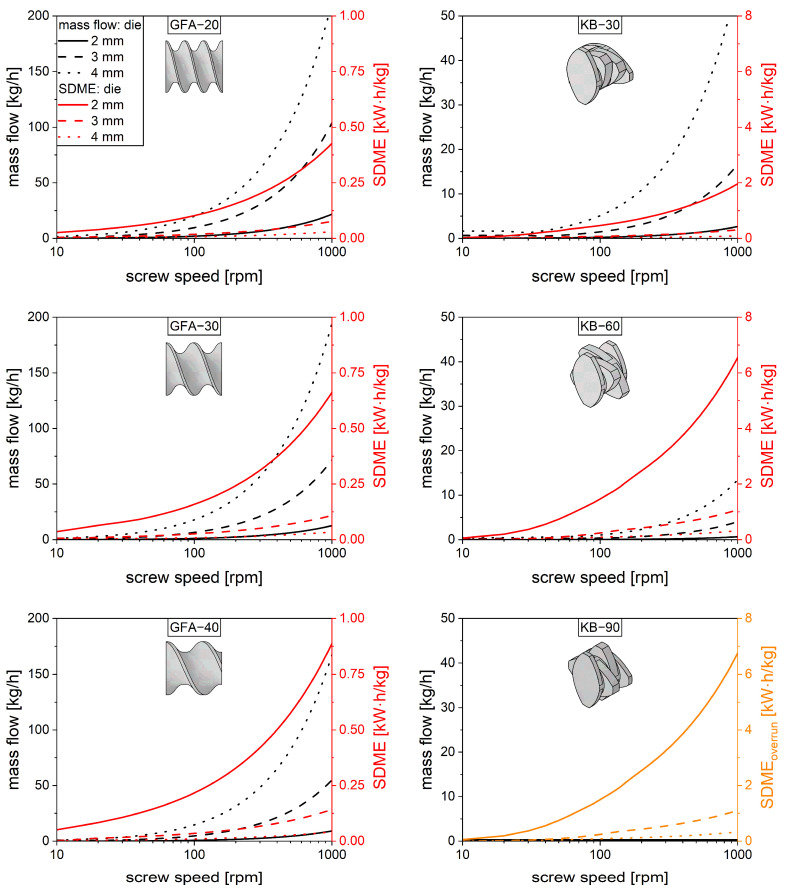
Mass flow (black lines) and specific dissipated mechanical energy (SDME) (red/orange lines) as a function of screw speed for typical die diameters (2, 3, 4 mm). Conveying elements (pitches: 20, 30 and 40 mm) (**left**) and kneading elements (staggering angles: 30, 60, and 90°) (**right**). SDME_overrun_ of KB-90 (orange lines) with overrun throughput of KB-60.

For purposes of discussion, a copovidone melt of 180 °C was considered, which is frequently used in hot-melt extrusion processes [42,43]. The mass flow rate and the power dissipation were calculated for a 30 mm length of conveying and kneading elements, with consideration of various throughputs (die diameters) and screw speeds, while an isothermal process was assumed. The required parameters such as polymer rheology and die length were taken from the literature [44]. The throughput was calculated using Equations (1) and (9). The mandatory die pressure was calculated based on the Hagen–Poiseuille law coupled to the Weissenberg–Rabinowitsch correlation [44] (Equations (8) and (9)). The system of equations was solved numerically for the volume flow as a function of screw speed using different die diameters (Figure 8). Based on this, the power of a screw element $\left(P\right)$ was split up in terms of power related to pressure $\left(P_{∆p}\right)$ and dissipated power $\left(P_{dis}\right)$ (Equation (13)). Thereby, the pressure power is expressed as the product of the pressure difference $\left(∆p\right)$ and the volume flow $\left(\dot{V}\right)$.

(13) $$P_{dis}={P-P}_{∆p}=P-∆p\cdot \dot{V}$$

The dissipated power can be viewed as the power accessible for processing the material in a twin-screw extrusion process, which is a function of screw speed as well as die diameter (Figure 8). In the interests of comparability, the power $\left(P_{dis}\right)$ is related to the mass flow $\left(\dot{m}\right)$, resulting in specific dissipated mechanical energy $\left(SDME\right)$. All of these calculations refer to a single filled screw element of 30 mm at isothermal conditions for the polymer copovidone.

For all conveying elements (Figure 8, left), the inherent throughput increased with the screw speed as well as the die diameter. Differences between the screw elements are visible but rather small. The lower pitch leads to higher throughput since the material is transported more efficiently, based on the higher surface area of the screw element. The dissipated power also increases with the screw speed, but it decreases with the die diameter, which is attributed to the pressure flow. The inherent throughput of the kneading elements (Figure 8, right) is much smaller compared to the conveying elements. This is in good agreement with the literature [6] and can be explained by the discontinuous change in pitch, which is less efficient for axial material transport. Higher staggering angles lead to less inherent throughput until zero is reached for the kneading element with the 90° staggering angle. Based on this, the specific dissipated mechanical energy will converge to infinity, since the material remains in the screw. In real extrusion processes, this element type will be overrun, leading to the volume flow being defined by neighboring elements. For visualization purposes, the specific dissipated mechanical energy for the 90° kneading block was calculated using the mass flow rate of the 60° kneading block in Figure 8. In this way, a similar energy dissipation was observed, which can be attributed to comparable parameters $\left(B_{2}\right)$ of both elements. Generally, higher staggering angles lead to higher energy dissipation, which is attributed to the initial lower throughput.

## 5. Conclusions

In this study, an experimental and modeling framework was established to determine the screw parameters for Newtonian and shear-thinning flow conditions to model hot-melt extrusion processes. Thus, three conveying elements varying in pitch and three kneading elements varying in staggering angle were characterized with respect to their conveying, pressure, and power performance using Newtonian and shear-thinning materials. The experiments were performed with a specially designed extruder test rig. With this, the experimentally determined Newtonian screw parameters ($A_{1},A_{2},B_{1},B_{2})$ were close to the measured and simulated parameters from the literature. Using shear-thinning materials, the screw parameters for shear-thinning behavior $\left(A_{3},B_{3}\right)$ can be obtained by means of linearization so linear regression analysis can be performed. Shear-thinning viscosity results in a significant reduction in pressure and power characteristics due to the shear rate dependence of the viscosity, which is quantitatively described with the screw parameters $\left(A_{3},B_{3}\right)$. Furthermore, the mechanical energy dissipation could be determined with these models, offering enormous potential for modeling product quality and energy consumption. By utilizing the six geometry parameters determined in this study, an entire hot-melt extrusion process can now be modeled in 1D and improved through in silico screw optimization.

## Figures and Tables

**Table 1:** Material constants for describing the viscosity of silicone oil with the Newtonian approach, and silicone rubber and HEC solution with the Carreau approach. Reference temperature $T_{ref}$ = 25 °C for Arrhenius approach as $\bar{x}\pm s$ (n = 3). The densities were taken from the literature [34,35,36].

	Silicone Oil	Silicone Rubber	HEC Solution
$\eta_{0}\;[Pa\cdot s]$	96 ± 0.1	224 ± 11	107 ± 1
${\dot{\gamma }}_{c}\;[s^{-1}]$	-	0.043 ± 0.004	0.260 ± 0.007
c [−]	-	0.316 ± 0.003	0.598 ± 0.004
$E_{A}\;[kJ\cdot {kg}^{-1}]$	14,327 ± 218	24,512 ± 4206	39,569 ± 695
$T_{ref}\;\left[{}^{\circ}C\right]$	25	25	25
$\rho \;[kg\cdot m^{-3}]$	973 [34]	1070 [35]	1008 [36]

**Table 2:** Dimensionless screw parameters of conveying elements for Newtonian flow $\left(A_{1},A_{2}\right)$; shear-thinning flow $\left(A_{3}\right)$ as pressure characteristics and Newtonian flow $\left(B_{1},B_{2}\right)$; and shear-thinning flow $\left(B_{3}\right)$ as power characteristics by linear regression and the confidence interval. $\bar{x}$ ± CI (α = 0.05), in comparison to the literature [6,29]. The pitch of the conveying elements was normalized to the screw diameter. Not available data is given as n/a.

	Measured Conveying Elements			König [6]			Eitzlmayr [29]	
Label	GFA-20	GFA-30	GFA-40	-	-	-	16/16	24/24
Pitch/d [-]	0.70	1.05	1.40	0.70	1.05	1.40	0.89	1.33
A_1_	0.210 ± 0.007	0.319 ± 0.011	0.406 ± 0.013	0.203	0.319	0.416	0.2257	0.3593
A_2_	4278 ± 55	2677 ± 29	1866 ± 15	4437	2545	1956	808.6	766.5
A_3_	37.60 ± 1.86	28.62 ± 0.77	18.45 ± 0.44	n/a	n/a	n/a	41.49	39.33
B_1_	0.443 ± 0.035	0.702 ± 0.071	0.947 ± 0.033	0.722	1.040	1.384	n/a	n/a
B_2_	4039 ± 75	3041 ± 56	2677 ± 12	3085	2737	2719	n/a	n/a
B_3_	82.70 ± 7.81	44.15 ± 1.24	37.88 ± 0.97	n/a	n/a	n/a	n/a	n/a

**Table 3:** Dimensionless screw parameters of kneading elements for Newtonian flow $\left(A_{1},A_{2}\right)$; shear-thinning flow $\left(A_{3}\right)$ as pressure characteristics and Newtonian flow $\left(B_{1},B_{2}\right)$; shear-thinning flow $\left(B_{3}\right)$ as power characteristics by linear regression and the confidence interval. $\bar{x}$ ± CI (α = 0.05), in comparison to the literature [10,29]. The staggering angle of the kneading elements was normalized to the screw diameter. Not available data is given as n/a.

	Measured Kneading Elements			Stritzinger [10]			Eitzlmayr [29]
Label	KB-30	KB-60	KB-90	30°	60°	90°	KB 45/5/8
$\mathrm{Angle}\;\left[{}^{\circ}\right]$ $/d\;\left[mm\right]$	1.05	2.11	3.16	1.03	2.05	3.08	2.5
A_1_	0.352 ± 0.028	0.059 ± 0.01	→ 0	0.444	0.107	n/a	0.1545
A_2_	818 ± 13	220 ± 12	→ 0	826	280	1.83	259.1
A_3_	17.02 ± 1.21	5.14 ± 0.59	→ 0	n/a	n/a	n/a	13.29
B_1_	1.53 ± 0.153	10.834 ± 3.455	→∞	2.131	3.262	n/a	n/a
B_2_	1834 ± 10	1407 ± 13	1358 ± 28	3518	2453	2775	n/a
B_3_	42.43 ± 1.89	27.89 ± 0.91	22.63 ± 1.73	n/a	n/a	n/a	n/a

## Data Availability

The raw data supporting the conclusions of this article are included in the Supplementary Materials.

## Supplementary Materials

The following supporting information can be downloaded at https://www.mdpi.com/article/10.3390/pharmaceutics17030353/s1. Raw data of viscosity measurements and extrusion screw experiments were provided.

## Abbreviations

List of Symbols and Abbreviations.

Latin Symbols	Definition	Unit
$A_{1},A_{2}$	dimensionless pressure parameters	−
$B_{1},B_{2}$	dimensionless power parameters	−
$A_{3},B_{3}$	dimensionless shear parameters	−
$a_{T}$	temperature shift factor	−
CL	centerline distance	m
c	flow index	−
d	barrel diameter	m
$d_{a}$	screw outer diameter	m
$d_{i}$	screw inner diameter	m
$E_{A}$	Arrhenius activation energy	$J\cdot {mol}^{-1}$
l	axial length	m
M	torque	N·m
n	screw speed	$s^{-1}$
P	power	$J\cdot s^{-1}$
$P^{*}$	dimensionless power	−
p	pressure	Pa
$p^{*}$	dimensionless pressure	−
R	ideal gas constant	$J\cdot {mol}^{-1}\cdot K^{-1}$
T	temperature	K
$T_{ref}$	reference temperature	K
$\dot{V}$	volume flow	$m^{3}\cdot s^{-1}$
${\dot{V}}^{*}$	dimensionless volume flow	−
w	weight	kg
Greek symbols	definition	unit
$\dot{\gamma }$	shear rate	$s^{-1}$
${\dot{\gamma }}_{c}$	critical shear rate	$s^{-1}$
${\dot{\gamma }}_{r}$	representative shear rate	$s^{-1}$
∆	difference	−
η	viscosity	Pa·s
$\eta_{0}$	zero shear rate viscosity	Pa·s
$\eta \left(\dot{\gamma }\right)$	dynamic viscosity	Pa·s
Λ	conveying parameter	−
Abbreviations	definition	
1D	one-dimensional	
3D	three-dimensional	
HEC	hydroxyethylcellulose	
Note: Tradenames and trademarks are used without particular labeling.		

## Appendix A

**Table A1:** Dimensions of the Leistritz ZSE 27 MAXX extruder and the conveying and kneading elements. The *-marked values are measured at the equipment and the others are given by the manufacturer.

Dimension	Symbol	Value
diameter ratio	$d_{a}/d_{i}\;[-]$	1.66
conveying element screw diameter	$d_{a}\;[mm]$	28.25 *
kneading element screw diameter	$d_{a}\;[mm]$	27.97 *
barrel diameter	$d\;\left[mm\right]$	28.45 *
centerline distance	CL [mm]	23
conveying element length	l [mm]	30
kneading element length	l [mm]	15
pitch	[mm]	20, 30, 40
staggering angle	$\alpha \;\left[{}^{\circ}\right]$	30, 60, 90
